# (4-Aza-1-azoniabicyclo­[2.2.2]octane-κ*N* ^4^)trichloridocobalt(II)

**DOI:** 10.1107/S1600536812017199

**Published:** 2012-04-25

**Authors:** Qinqin Zhou, Bo-Han Zhu

**Affiliations:** a Ordered Matter Science Research Center, Southeast University, Nanjing 211189, People’s Republic of China

## Abstract

In the title compound, [CoCl_3_(C_6_H_13_N_2_)], the tetra­hedrally coordinated Co^II^ ion has Co—Cl distances ranging from 2.2220 (11) to 2.2449 (9) Å and a Co—N distance of 2.056 (2) Å. In the crystal, N—H⋯Cl hydrogen bonds link mol­ecules into chains in [010]. Weak C—H⋯Cl inter­actions stabilize further the crystal packing.

## Related literature

For background to related ferroelectric materials, see: Fu *et al.* (2010 ▶); Zhang *et al.* (2008 ▶). For the crystal structure of the Zn analogue of the title compound, see: Wei & Willett (2001 ▶).
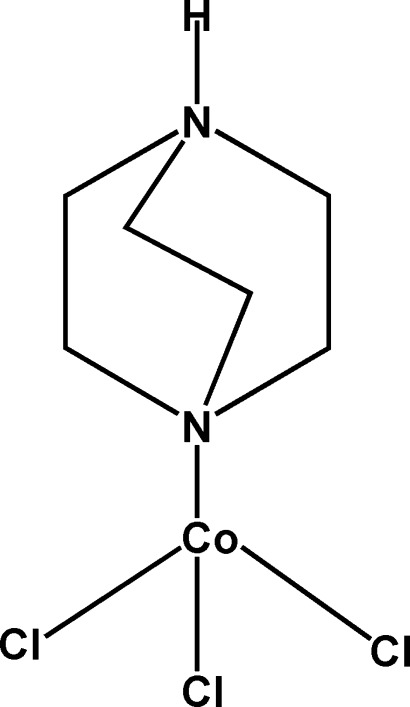


## Experimental

### 

#### Crystal data

[CoCl_3_(C_6_H_13_N_2_)]
*M* *_r_* = 278.46
Monoclinic, 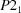

*a* = 6.6873 (13) Å
*b* = 12.433 (3) Å
*c* = 6.9298 (14) Å
β = 116.96 (3)°
*V* = 513.6 (2) Å^3^
*Z* = 2
Mo *K*α radiation
μ = 2.40 mm^−1^
*T* = 293 K
0.36 × 0.32 × 0.28 mm

#### Data collection

Rigaku SCXmini diffractometer
Absorption correction: multi-scan (*CrystalClear*; Rigaku, 2005 ▶) *T*_min_ = 0.438, *T*_max_ = 0.511
5285 measured reflections
2343 independent reflections
2263 reflections with *I* > 2σ(*I*)
*R*_int_ = 0.029

#### Refinement

*R*[*F* ^2^ > 2σ(*F* ^2^)] = 0.024
*wR*(*F* ^2^) = 0.054
*S* = 1.06
2343 reflections
113 parameters
1 restraint
H atoms treated by a mixture of independent and constrained refinement
Δρ_max_ = 0.29 e Å^−3^
Δρ_min_ = −0.33 e Å^−3^
Absolute structure: Flack (1983 ▶), 1103 Friedel pairs
Flack parameter: 0.032 (13)

### 

Data collection: *CrystalClear* (Rigaku, 2005 ▶); cell refinement: *CrystalClear*; data reduction: *CrystalClear*; program(s) used to solve structure: *SHELXS97* (Sheldrick, 2008 ▶); program(s) used to refine structure: *SHELXL97* (Sheldrick, 2008 ▶); molecular graphics: *SHELXTL* (Sheldrick, 2008 ▶); software used to prepare material for publication: *SHELXL97*.

## Supplementary Material

Crystal structure: contains datablock(s) I, global. DOI: 10.1107/S1600536812017199/cv5276sup1.cif

Structure factors: contains datablock(s) I. DOI: 10.1107/S1600536812017199/cv5276Isup2.hkl

Additional supplementary materials:  crystallographic information; 3D view; checkCIF report

## Figures and Tables

**Table 1:** Hydrogen-bond geometry (Å, °)

*D*—H⋯*A*	*D*—H	H⋯*A*	*D*⋯*A*	*D*—H⋯*A*
N1—H1⋯Cl1^i^	0.89 (3)	2.37 (3)	3.217 (2)	160 (3)
C3—H3*A*⋯Cl2^ii^	0.97	2.83	3.603 (3)	137
C3—H3*B*⋯Cl3^i^	0.97	2.72	3.621 (3)	155
C6—H6*A*⋯Cl3^iii^	0.97	2.81	3.494 (3)	129
C6—H6*A*⋯Cl2^iv^	0.97	2.82	3.605 (3)	139
C5—H5*B*⋯Cl2^v^	0.97	2.82	3.735 (3)	158

Symmetry codes: (i) 
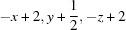
; (ii) 
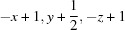
; (iii) 
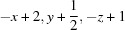
; (iv) 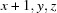; (v) 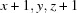.

**Fig. 1.**
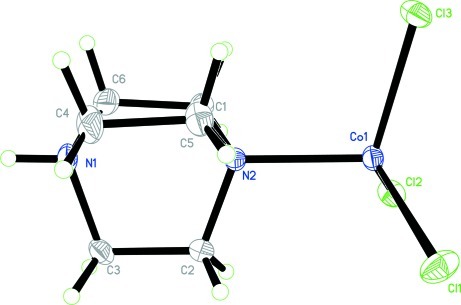
The molecular structure of the title compound, showing the atomic numbering scheme with 30% probability displacement ellipsoids.

## supplementary crystallographic information

### Comment

The study of ferroelectric materials has received much attention and some materials have predominantly dielectric-ferroelectric performance (Fu *et al*, 2010; Zhang *et al.*, 2008). The title compound, (I), was prepared in an attempt to obtain analogs to Zn(dabcoH)Cl_3_ (Wei & Willett, 2001).

In (I) (Fig. 1), the tetrahedrally coordinated Co^II^ ion has Co—Cl distances ranging from 2.2224 (11) to 2.2449 (9)Å and a Co—N distance of 2.057 (2) Å. In the crystal structure, intermolecular N—H···Cl hydrogen bonds (Table 1) link molecules into chains in [010]. Weak C—H···Cl interactions (Table 1) stabilize further the crystal packing.

### Experimental

(dabcoH)Cl(10 mmol, 1.48 g) was dissolved in 15 mL water, then CoCl_2_^.^6H_2_O (10 mmol, 2.38 g) in 15 ml water was added and the mixed solution was filtered. After a few days, black microcrystasls were obtained by slow evaporation at room temperature in air.

### Refinement

N-bound atom H1 was located on a difference map and isotropically refined. C-bound H atoms were placed in calculated positions (C—H = 0.97 Å), and refined as riding with *U*_iso_(H) = 1.2-1.5 *U*_eq_(C).

### Crystal data

**Table:**

[CoCl_3_(C_6_H_13_N_2_)]	*F*(000) = 282
*M**_r_* = 278.46	*D*_x_ = 1.801 Mg m^−^^3^
Monoclinic, *P*2_1_	Mo *K*α radiation, λ = 0.71073 Å
Hall symbol: P 2yb	Cell parameters from 1860 reflections
*a* = 6.6873 (13) Å	θ = 3.3–27.5°
*b* = 12.433 (3) Å	µ = 2.40 mm^−^^1^
*c* = 6.9298 (14) Å	*T* = 293 K
β = 116.96 (3)°	Block, black
*V* = 513.6 (2) Å^3^	0.36 × 0.32 × 0.28 mm
*Z* = 2	

### Data collection

**Table:**

Rigaku SCXmini diffractometer	2343 independent reflections
Radiation source: fine-focus sealed tube	2263 reflections with *I* > 2σ(*I*)
Graphite monochromator	*R*_int_ = 0.029
Detector resolution: 13.6612 pixels mm^-1^	θ_max_ = 27.5°, θ_min_ = 3.3°
ω scans	*h* = −8→8
Absorption correction: multi-scan (*CrystalClear*; Rigaku, 2005)	*k* = −16→16
*T*_min_ = 0.438, *T*_max_ = 0.511	*l* = −8→8
5285 measured reflections	

### Refinement

**Table:**

Refinement on *F*^2^	Secondary atom site location: difference Fourier map
Least-squares matrix: full	Hydrogen site location: inferred from neighbouring sites
*R*[*F*^2^ > 2σ(*F*^2^)] = 0.024	H atoms treated by a mixture of independent and constrained refinement
*wR*(*F*^2^) = 0.054	*w* = 1/[σ^2^(*F*_o_^2^) + (0.0178*P*)^2^] where *P* = (*F*_o_^2^ + 2*F*_c_^2^)/3
*S* = 1.06	(Δ/σ)_max_ = 0.015
2343 reflections	Δρ_max_ = 0.29 e Å^−^^3^
113 parameters	Δρ_min_ = −0.33 e Å^−^^3^
1 restraint	Absolute structure: Flack (1983), 1103 Friedel pairs
Primary atom site location: structure-invariant direct methods	Flack parameter: 0.032 (13)

### Special details

**Table:**

Geometry. All e.s.d.'s (except the e.s.d. in the dihedral angle between two l.s. planes) are estimated using the full covariance matrix. The cell e.s.d.'s are taken into account individually in the estimation of e.s.d.'s in distances, angles and torsion angles; correlations between e.s.d.'s in cell parameters are only used when they are defined by crystal symmetry. An approximate (isotropic) treatment of cell e.s.d.'s is used for estimating e.s.d.'s involving l.s. planes.
Refinement. Refinement of *F*^2^ against ALL reflections. The weighted *R*-factor *wR* and goodness of fit *S* are based on *F*^2^, conventional *R*-factors *R* are based on *F*, with *F* set to zero for negative *F*^2^. The threshold expression of *F*^2^ > σ(*F*^2^) is used only for calculating *R*-factors(gt) *etc*. and is not relevant to the choice of reflections for refinement. *R*-factors based on *F*^2^ are statistically about twice as large as those based on *F*, and *R*- factors based on ALL data will be even larger.

### Fractional atomic coordinates and isotropic or equivalent isotropic displacement parameters (Å^2^)

**Table:**

	*x*	*y*	*z*	*U*_iso_*/*U*_eq_	
Co1	0.56604 (4)	−0.11682 (3)	0.70822 (5)	0.02232 (9)	
Cl2	0.22414 (9)	−0.06559 (5)	0.46983 (10)	0.03324 (15)	
Cl3	0.71041 (10)	−0.24845 (5)	0.59195 (11)	0.03334 (15)	
Cl1	0.58299 (12)	−0.15466 (6)	1.03206 (12)	0.04149 (18)	
C3	0.8485 (4)	0.2022 (2)	0.8668 (4)	0.0297 (6)	
H3A	0.7499	0.2592	0.7795	0.036*	
H3B	0.9268	0.2270	1.0155	0.036*	
C6	0.8946 (5)	0.1423 (2)	0.5520 (5)	0.0376 (7)	
H6A	1.0015	0.1210	0.5004	0.045*	
H6B	0.8083	0.2026	0.4658	0.045*	
N2	0.7709 (3)	0.01327 (16)	0.7478 (3)	0.0192 (4)	
C1	0.7407 (4)	0.0495 (2)	0.5324 (4)	0.0263 (5)	
H1A	0.5861	0.0715	0.4455	0.032*	
H1B	0.7714	−0.0100	0.4591	0.032*	
C5	1.0080 (4)	−0.0144 (2)	0.8778 (5)	0.0315 (6)	
H5A	1.0497	−0.0698	0.8043	0.038*	
H5B	1.0302	−0.0429	1.0164	0.038*	
C2	0.7144 (4)	0.1024 (2)	0.8533 (4)	0.0292 (6)	
H2A	0.7435	0.0807	0.9982	0.035*	
H2B	0.5557	0.1185	0.7726	0.035*	
N1	1.0141 (4)	0.17317 (18)	0.7836 (4)	0.0359 (6)	
C4	1.1569 (4)	0.0829 (2)	0.9146 (5)	0.0417 (8)	
H4A	1.2289	0.1021	1.0669	0.050*	
H4B	1.2726	0.0672	0.8709	0.050*	
H1	1.107 (5)	0.227 (3)	0.801 (5)	0.054 (9)*	

### Atomic displacement parameters (Å^2^)

**Table:**

	*U*^11^	*U*^22^	*U*^33^	*U*^12^	*U*^13^	*U*^23^
Co1	0.02136 (15)	0.01775 (14)	0.02842 (17)	−0.00118 (13)	0.01178 (13)	0.00032 (14)
Cl2	0.0236 (3)	0.0317 (3)	0.0374 (4)	0.0042 (3)	0.0077 (3)	−0.0033 (3)
Cl3	0.0330 (3)	0.0271 (3)	0.0387 (4)	0.0038 (3)	0.0151 (3)	−0.0067 (3)
Cl1	0.0459 (4)	0.0486 (4)	0.0399 (4)	0.0150 (3)	0.0282 (3)	0.0172 (3)
C3	0.0292 (13)	0.0218 (12)	0.0389 (16)	−0.0013 (10)	0.0162 (12)	−0.0093 (11)
C6	0.0531 (18)	0.0252 (13)	0.0506 (19)	−0.0018 (12)	0.0375 (16)	0.0032 (13)
N2	0.0179 (9)	0.0177 (9)	0.0221 (11)	−0.0009 (7)	0.0092 (9)	0.0003 (8)
C1	0.0314 (13)	0.0262 (13)	0.0213 (13)	−0.0019 (11)	0.0119 (11)	0.0034 (11)
C5	0.0211 (12)	0.0267 (14)	0.0396 (16)	0.0022 (10)	0.0075 (12)	0.0023 (12)
C2	0.0350 (13)	0.0240 (13)	0.0374 (15)	−0.0030 (10)	0.0240 (13)	−0.0069 (11)
N1	0.0307 (12)	0.0215 (11)	0.0629 (17)	−0.0090 (9)	0.0278 (12)	−0.0081 (11)
C4	0.0169 (12)	0.0366 (16)	0.061 (2)	−0.0037 (11)	0.0088 (13)	−0.0122 (15)

### Geometric parameters (Å, º)

**Table:**

Co1—N2	2.0559 (19)	N2—C2	1.468 (3)
Co1—Cl2	2.2220 (11)	N2—C1	1.483 (3)
Co1—Cl3	2.2281 (7)	C1—H1A	0.9700
Co1—Cl1	2.2449 (9)	C1—H1B	0.9700
C3—N1	1.506 (3)	C5—C4	1.513 (3)
C3—C2	1.509 (3)	C5—H5A	0.9700
C3—H3A	0.9700	C5—H5B	0.9700
C3—H3B	0.9700	C2—H2A	0.9700
C6—N1	1.483 (4)	C2—H2B	0.9700
C6—C1	1.511 (3)	N1—C4	1.486 (4)
C6—H6A	0.9700	N1—H1	0.89 (3)
C6—H6B	0.9700	C4—H4A	0.9700
N2—C5	1.466 (3)	C4—H4B	0.9700
			
N2—Co1—Cl2	105.80 (6)	N2—C1—H1B	109.3
N2—Co1—Cl3	104.73 (6)	C6—C1—H1B	109.3
Cl2—Co1—Cl3	114.22 (3)	H1A—C1—H1B	108.0
N2—Co1—Cl1	107.64 (6)	N2—C5—C4	111.2 (2)
Cl2—Co1—Cl1	111.83 (4)	N2—C5—H5A	109.4
Cl3—Co1—Cl1	111.95 (3)	C4—C5—H5A	109.4
N1—C3—C2	107.37 (19)	N2—C5—H5B	109.4
N1—C3—H3A	110.2	C4—C5—H5B	109.4
C2—C3—H3A	110.2	H5A—C5—H5B	108.0
N1—C3—H3B	110.2	N2—C2—C3	111.83 (19)
C2—C3—H3B	110.2	N2—C2—H2A	109.2
H3A—C3—H3B	108.5	C3—C2—H2A	109.2
N1—C6—C1	107.8 (2)	N2—C2—H2B	109.2
N1—C6—H6A	110.1	C3—C2—H2B	109.2
C1—C6—H6A	110.1	H2A—C2—H2B	107.9
N1—C6—H6B	110.1	C6—N1—C4	110.3 (2)
C1—C6—H6B	110.1	C6—N1—C3	110.3 (2)
H6A—C6—H6B	108.5	C4—N1—C3	109.1 (2)
C5—N2—C2	108.7 (2)	C6—N1—H1	110 (2)
C5—N2—C1	107.81 (17)	C4—N1—H1	106 (2)
C2—N2—C1	108.89 (19)	C3—N1—H1	111 (2)
C5—N2—Co1	111.54 (15)	N1—C4—C5	108.0 (2)
C2—N2—Co1	110.73 (14)	N1—C4—H4A	110.1
C1—N2—Co1	109.05 (14)	C5—C4—H4A	110.1
N2—C1—C6	111.4 (2)	N1—C4—H4B	110.1
N2—C1—H1A	109.3	C5—C4—H4B	110.1
C6—C1—H1A	109.3	H4A—C4—H4B	108.4

### Hydrogen-bond geometry (Å, º)

**Table:**

*D*—H···*A*	*D*—H	H···*A*	*D*···*A*	*D*—H···*A*
N1—H1···Cl1^i^	0.89 (3)	2.37 (3)	3.217 (2)	160 (3)
C3—H3*A*···Cl2^ii^	0.97	2.83	3.603 (3)	137
C3—H3*B*···Cl3^i^	0.97	2.72	3.621 (3)	155
C6—H6*A*···Cl3^iii^	0.97	2.81	3.494 (3)	129
C6—H6*A*···Cl2^iv^	0.97	2.82	3.605 (3)	139
C5—H5*B*···Cl2^v^	0.97	2.82	3.735 (3)	158

Symmetry codes: (i) −*x*+2, *y*+1/2, −*z*+2; (ii) −*x*+1, *y*+1/2, −*z*+1; (iii) −*x*+2, *y*+1/2, −*z*+1; (iv) *x*+1, *y*, *z*; (v) *x*+1, *y*, *z*+1.
